# Estimating antibiotics consumption in a tertiary care hospital in Islamabad using a WHO’s defined daily dose methodology

**DOI:** 10.1186/s13756-023-01311-2

**Published:** 2023-11-23

**Authors:** Naila Jabeen, Waseem Ullah, Javeria Khalid, Zia Samad

**Affiliations:** 1 Department of Pharmacy Practice, Shifa College of Pharmaceutical Sciences, Shifa Tameer-e-Millat University, Islamabad, Pakistan; 2 University of Houston, Houston, TX USA; 3 National Tuberculosis Control Program, Islamabad Capital Territory, Islamabad, Pakistan

**Keywords:** Monitoring antibiotic usage, Consumption variation, Inpatient care, Defined daily dose, Antibiotic resistance, And antibiotic stewardship

## Abstract

**Background:**

Antibiotics have helped to reduce the incidence of common infectious diseases in all modern healthcare systems, but improper use of antibiotics including their overuse and misuse can change the bacteria so much that antibiotics don’t work against them. In case of developing imposable selective pressure with regard to the proportion of hospitalized patients who receive antibiotics, the quantity of antibiotics that are prescribed to them, and the proportion of patients who receive antibiotic treatment is one of the major contributors to the rising global health issue of antimicrobial resistance. Concerning the levels of antibiotic consumption in Pakistani hospitals, there is negligible research data available.

**Aim:**

This study aimed to evaluate five-year inpatient antibiotic use in a tertiary care hospital in Islamabad using the World Health Organization (WHO) Recommended Anatomical Therapeutic Chemical (ATC) Classification / Defined Daily Dose (DDD) methodology.

**Method:**

It was a descriptive study involving a retrospective record review of pharmacy records of antibiotics dispensed (amount in grams) to patients across different specialties of the hospital from January 2017 to December 2021 (i.e., 60 consecutive months). The antibiotic consumption was calculated by using the DDD/100-Bed Days (BDs) formula, and then relative percent change was estimated using Microsoft Excel 2021 edition.

**Result:**

A total of 148,483 (77%) patients who received antibiotics were included in the study out of 193,436 patients admitted in the hospital. Antibiotic consumption trends showed considerable fluctuations over a five-year period. It kept on declining irregularly from 2017 to 2019, inclined vigorously in 2020, and then suddenly dropped to the lowest DDD/100 BDs value (96.02) in the last year of the study. The overall percentage of encounters in which antibiotics were prescribed at tertiary care hospital was 77% which is very high compared to the WHO standard reference value (< 30%). WATCH group antibiotics were prescribed (76%) and consumed more within inpatient settings than Access (12%) and Reserve (12%) antibiotics.

**Conclusion:**

The hospital antibiotic consumption data is well maintained across different inpatient specialties but it is largely non-aligned with WHO AWaRe (Access-Watch-Reserve) antibiotics use and optimization during 2017–2021. Compared to the WHO standard reference figure, the overall percentage of antibiotics encountered was higher by about 47%. Antibiotic consumption trends vary with a slight increase in hospital occupancy rate, with positive relative changes being lower in number but higher in proportion than negative changes. Although the hospital antibiotics policy is in place but seems not to be followed with a high degree of adherence.

## Background

In all health systems so far, antibiotics have lowered the prevalence of common infectious diseases and are indispensable in countless medical interventions [1]. But inappropriate consumption (overuse and misuse) of antibiotics is a key driver of the growing global health threat of antimicrobial resistance (AMR) [2]. AMR makes illnesses harder to cure and raises the risk of disease transmission, life-threatening sickness, and even death. These consequences can result in resistance to the antibiotics’ mode of action and diseases continue to exist in the body, raising the chance of infection spreading to other people [3]. While antibiotic consumption mainly focuses on imposable selective pressure with relevance to the percentage of hospitalized patients put on antibiotics, the number of antibiotics prescribed to them as well as the number of patients getting treated with the use of antibiotics [4].

In order to optimize antibiotic consumption, the WHO Essential Medicines List (EML) Working Group developed a tool termed the AwaRe classification of antibiotics in 2017 and revised it in 2021 [5]. The classification includes following antibiotic groups; Access, Watch, Reserve, and Not recommended. Access group includes narrow-spectrum antibiotics generally recommended as 1st and 2nd choice agents for commonly encountered clinical infections. Watch group contains the most essential broad-spectrum antibiotics among the vitally important antimicrobials, coupled with those with a higher resistance potential. The antibiotics reserved for targeted use in multi-drug resistant infections as “last resort” options make it to the list of Reserve group of AwaRe classification, and “not recommended” group includes the combination of those antibiotics which have same spectrum of same infection coverage and shouldn’t be use together [6].

At individual hospitals, the connection between antibiotic use and resistance is widely understood across temporal and spatial scales [7], primary care facilities [8], nursing homes [9], communities [10], and across countries [11]. To counter this association, AMR national action plans have been developed by most of the countries that aim reducing antibiotic consumption per capita [12]. The member states of WHO including Pakistan have endorsed the antimicrobial resistant Global Action Plan (GAP) [13] with a promise that all nations will gather and submit statistics on antibiotic usage [14]. In Pakistan, the first real effort to achieve this Global Action Plan was done by launching a ‘National Framework’ to strategize the containment of AMR in 2016 followed by translating this framework into the country’s first AMR National Action Plan (NAP) in 2017 [15]. NAP operates through the National Institute of Health (NIH) as the national focal point as well as Provincial AMR focal persons for implementation of selected technical areas of workforce development, surveillance and response, laboratory system with relevance to AMR persons. Furthermore NIH announced to have mandatory antimicrobial stewardship launch in all hospitals of capital and also recently the AWaRe classification was added in the national essential list of medicines [16]. But still a strong framework and commitment needs to be fulfilled for the governance of AMR activities, policy dialogue and developments as well evidence-based decision making for establishing the dedicated provincial and federal setups for NAP implementation across the country [15]. Human antibiotic consumption in Pakistan is third highest in the world after China and India among lower-middle-income countries (LMIC) [12] Among high-income countries (HIC), United States, France, and Italy remain highest consumers of antibiotics. Between 2000 and 2015, In Pakistan the rate of antibiotic consumption grew from 16.2 to 19.6 DDDs per 1,000 people per day (21%) and from 0.8 to 1.3 billion daily defined doses (DDDs) (an increase of 65%) [12].

Despite the direct relationship of antibiotic consumption to resistance, and public health consequences of emerging resistant microorganism to the effects of antibiotics [17], limited evidence is available concerning the levels of antibiotic consumption in Pakistani hospitals inpatient (DDD/100 BDs) as well as out-patient settings (DDD/1000 inhibitant days) [18]. While multiple studies have reported the need for rational antibiotic prescribing practices to minimize the serious misuse of antibiotics, surprisingly, there are only a few published comparisons or descriptions of antibiotic consumption around the globe [19]. Hospitals can measure and compare their antibiotic consumption with other hospitals regardless of differences in antibiotic quantity and quality by estimating their antibiotic use in the form of Daily Defined Doses per 100 patient-days (DDD/100-BD). [20, 21]. In Pakistan at healthcare level only few studies have been conducted that focused on perception of healthcare professional about antibiotic stewardship, trend of antibiotic use, and pattern of antibiotic consumption using WHO’s prescribing indicators, respectively [22–24] Since no such information about the rates of antibiotic consumption in hospitals or wards in Islamabad using WHO ATC/DDD methodology has previously been made public, it is still unknown that how much antibiotics are being used in the capital territory.

### Aim

The study aimed to evaluate the five-year (between 2017 and 2021) inpatient antibiotic use in tertiary care hospital using the ATC/ DDD methodology.

## Method

### Research design

It was a descriptive study involving a retrospective record review of routinely maintained (aggregated quarterly surveillance) data of antibiotics dispensed to specialty wards of a tertiary care hospital.

### Study settings

The selected hospital site comprised 550 quaternary care beds (refers to the extension of tertiary care where advanced-level care is being provided to the patients) providing healthcare facilities to local and international patients along with 21 specialty care units in Islamabad. These specialty care units included Cardiology, Cardiac surgery, Emergency, Endocrinology, Ear Nose Throat (ENT), Gastroenterology, General Surgery, Liver transplant, Medical specialty, Nephrology, Neurology, Neurosurgery, Obstetrics/Gynecology, Oncology, Orthopedic, Pediatrics, Pediatric Surgery, Pulmonology, Rheumatology, Plastic surgery, and Urology.

### Inclusion/Exclusion Criteria

Antibiotics that fall under the ATC classification system [25], and were dispensed to admitted patients throughout the 5 years across in-patient specialties were included in the study. The antibiotics that fall under ATC classification but didn’t show 5 years consumption trend as well as those antibiotics that were dispensed to outpatient and home healthcare services were excluded from the study.

### Data collection

**Fig. 1:**
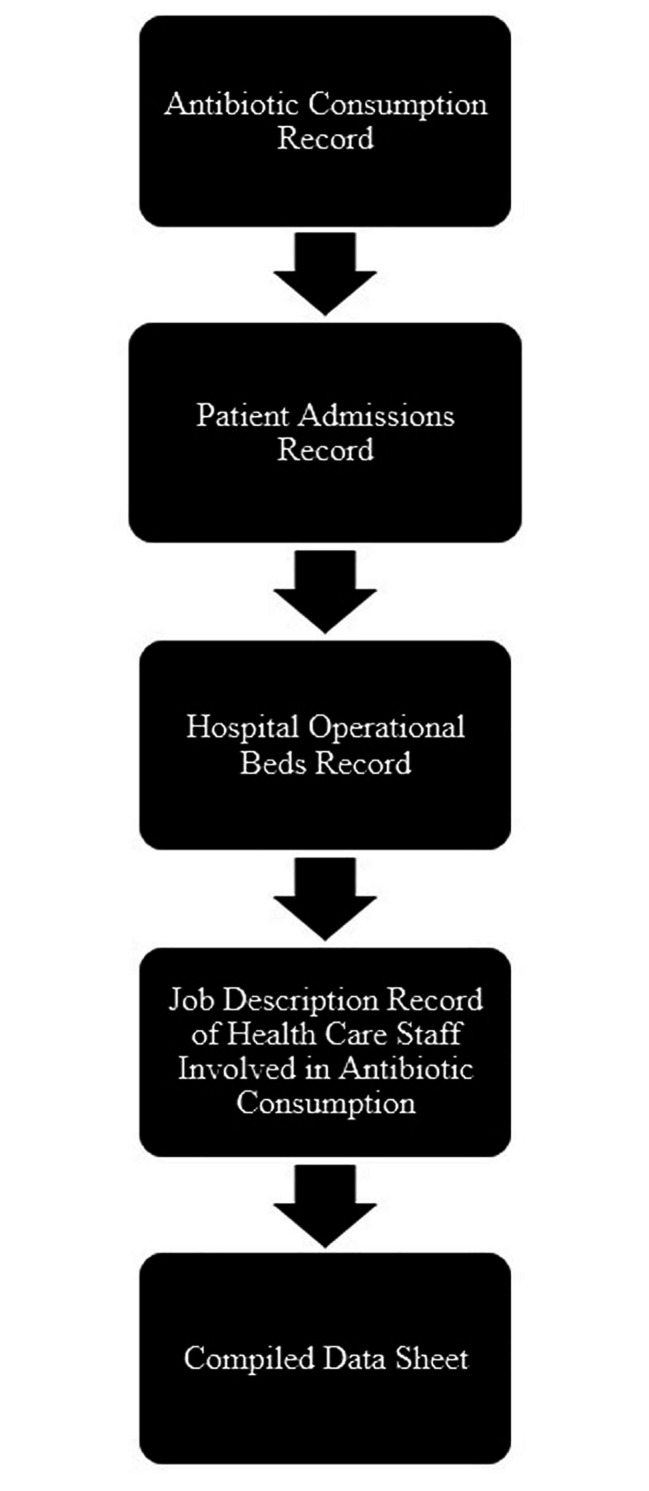
Steps involved in compiling raw data during data collection

Antibiotic consumption data for the selected years were extracted from the hospital’s pharmacy records with the help of HMIS (Health Management Information System). Consumption data included the antibiotics dispensed from 2017 to 2021 in form of an excel sheet containing specialty names, antibiotics (brand and generic name), dosage form, and amount of antibiotics (in gm or mg). Separate data of patient admission records for five years (2017–2021) was also extracted from the system with the help of the Information Technology (IT) department of the hospital. (See Figure 1)

Data of the total number of operational beds along with the number of patients on antibiotics among admitted patients (male and female) was extracted from HMIS with the help of the Quality department.

The conversations of data collection team with the clinical pharmacy staff about the antibiotics’ consumption were not formally recorded or documented, but data collectors were encouraged to diarize key findings.

### Calculation of antibiotic consumption

The WHO Collaborating Center for Drug Statistics Methodology advises using the ATC/DDD (The Anatomical Therapeutical Chemical Classification/ Defined Daily Doses System) classification [26]. The same method has been used to determine the amount of DDD for the various antibiotics for selected specialties.

### Data Analysis

**Fig. 2:**
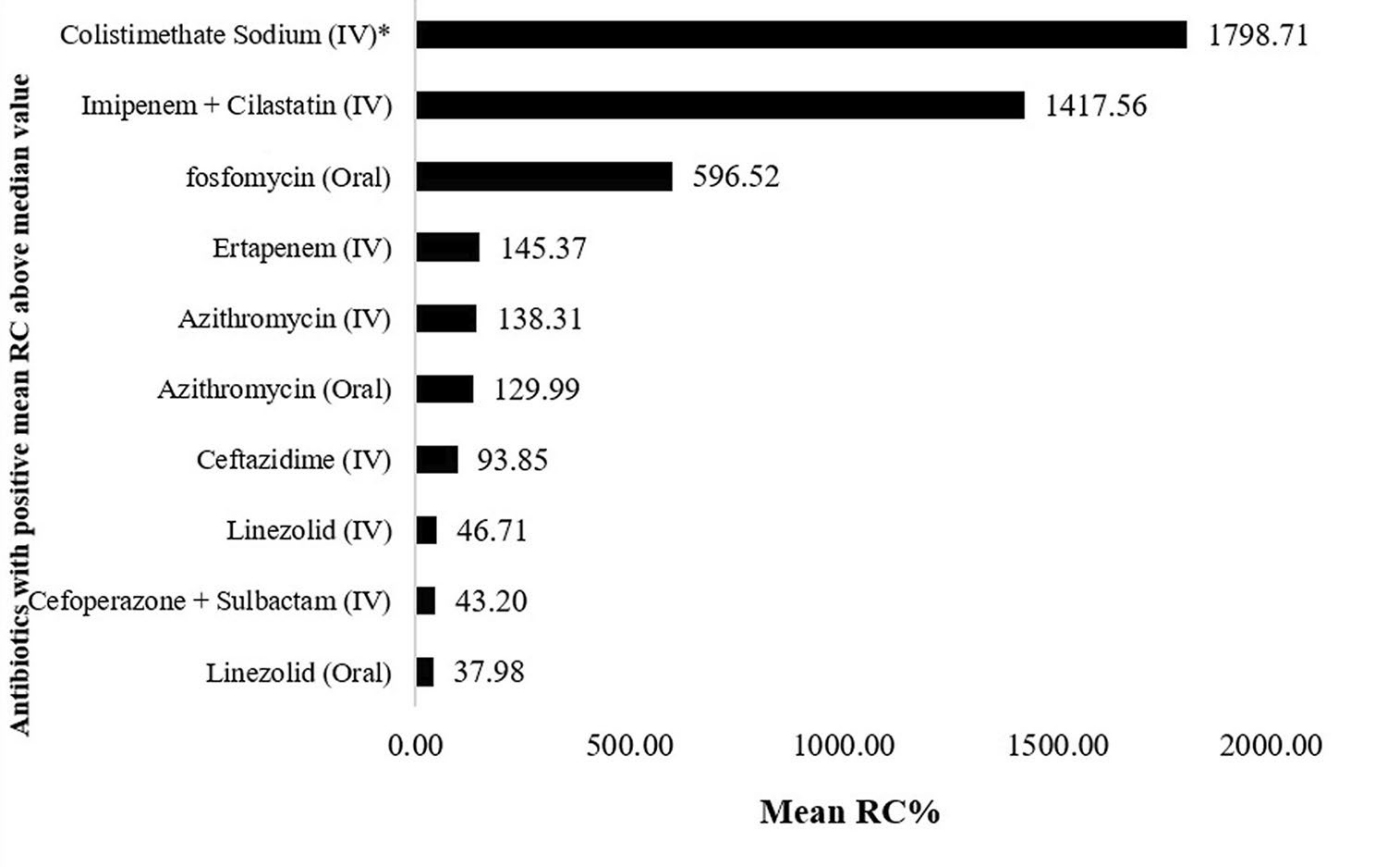
Proportion of positive mean relative change of anitibiotics consumed

Excel was used to organize, and clean the imported and calculated data (Microsoft Excel 2021 edition). The cleaned data was analyzed for input accuracy before being subjected to a descriptive analysis for the research period in order to determine the process indicators. The categorical variables were reported as counts and proportions (%) for descriptive (statistical) analysis. While a central tendency test (calculated by sorting the data in ascending order, finding the median, and choosing the drug with higher relative change above median) was followed for the figures to show the trend of increase and decrease in relative change of antibiotics consumption. (Figure 1 & 2)

## Results

A total of 148,483 (77%) patients who received antibiotics were included in the study out of 193,436 patients admitted during the study period (January 2017 to December 2021). The rate of antibiotic use (percentage of encounters with antibiotics) in the starting year of the study was 78.5% (27,287/34,750), dropped to 66.9% (29,759/44,433) in 2019 and then was extended up to highest mark of 84.4% (36,145/42,810) at the end of study. The hospital total bed capacity ranged from a minimum of 530 in 2017 to a maximum of 550 in 2021. While the bed occupancy rate showed a variation during first 3 years but remained constant in the last 2 years of study period. (See Table 1).

According to ATC as the classification system and DDD as a unit of measure, a total of 48 antibiotics made it to the inclusion criteria (AWaRe classification) whose mean relative change was calculated in this study. Out of them, 15 antibiotics belonged to Access group antibiotics (see Table 2), Watch group comprised 28 antibiotics (see Table 3), and only 5 antibiotics of the Reserve group (see Table 4) were prescribed and consumed among admitted patients across hospital specialties throughout the study period. The Relative change (RC) in form of percent was calculated keeping the first year (i.e., 2017) as standard and relative change of a further four years (2018–2021) to the initial standard year, adopted from a study conducted in Latin America [27].

Among the consumed (48 in number) AWaRe antibiotics, 20 antibiotics have a positive mean relative change which means their consumption has increased over the period of time and remaining 28 antibiotics have a negative mean relative change showing their consumption has decreased from 2017 to 2021.

Viewing the antibiotics with positive mean relative change, in order to provide the magnitude and summary measure of antibiotics consumption, a measure of central tendency test was undertaken [28]. So, after looking at the dispersion of data set of 20 antibiotics in our case, the median positive relative change of antibiotics was found to be 37.98% (range: 8.02-1798.91%). Therefore, out of 20, only 10 antibiotics were consumed higher than the median value mostly attributed by 3 antibiotics; Colistimethate sodium (IV) consumption increased (changed) by 1798.91% during the years 2018–2021 in comparison to its consumption in 2017, followed by Imipenem + Cilastatin (IV) and Fosfomycin (Oral) whose consumption trends respectively increased by 1417.56% and 596.52% during the study years. While lowest relative change was seen in case of Linezolid (Oral) whose consumption only increased by 37.98% from 2018 to 2021. Figure 2 highlight the distribution of increased consumption for the entire group of antibiotics with positive mean relative change depicted as proportion.

**Fig. 3:**
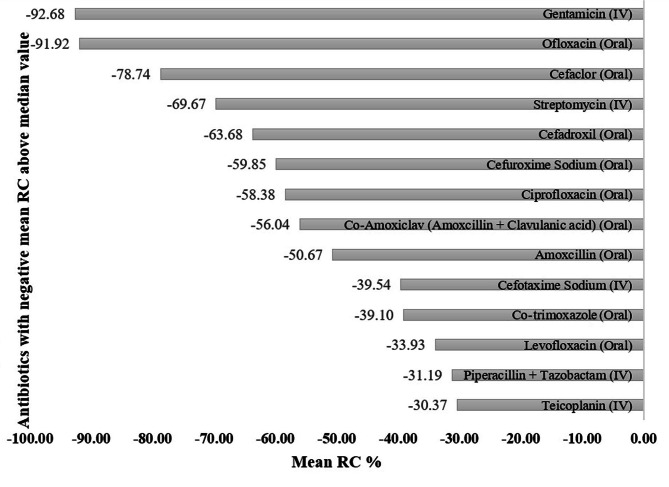
Proportion of negative mean relative change of anitibiotics consumed

Viewing the dispersion of data set of remaining 28 antibiotics whose consumption decreased from 2018 to 2021, the median negative relative change of antibiotics was found to be -28.46% (range: -92.68% **—** -30.27%). So, out of 28, only 14 antibiotics were consumed above than the median value mostly attributed by 3 antibiotics; Gentamicin (IV) consumption decreased (changed) by 92.68% during the years 2018–2021 in comparison to its consumption in 2017, closely followed by Ofloxacin (Oral) and Cefaclor (Oral) whose consumption trends respectively decreased by 91.92% and 78.74% during the study years. While lowest relative change was seen in case of Teicoplanin (IV) and Piperacillin + Tazobactam (IV), whose consumption only decreased by 30.37% and 31.19% from 2018 to 2021. Figure 3 highlights the distribution of decreased consumption for the entire group of antibiotics with negative mean relative change depicted as proportion.

## Discussion

This study is the first to estimate hospital-based antibiotic consumption at a regional (Islamabad Capital Territory) level in Pakistan, using ATC/DDD methodology and WHO AWaRe policy for international comparison. Since Our study quantified AWaRe-classified antibiotic consumption changes over a 5-year period, giving us the opportunity to benchmark the gaps between antibiotic utilization in a JCI-accredited hospital, which would be informing for Pakistan’s AMR national action plan, healthcare providers (physicians, pharmacists, nurses, microbiologists), decision-makers (hospital administration), researchers as well as the public utilizing the health services in this setting.

The overall percentage of encounters (for 5-years) in which antibiotics were prescribed at tertiary care hospital was 77% which is very high compared to the standard reference value (< 30%) of globally introduced WHO prescribing indicators [29]. This higher overall rate of antibiotic use might be due to the real or perceived high prevalence of antibiotic resistance, absence, or non-adherence to antibiotic treatment guidelines [30]. The former prevalence of antibiotics resistance can be confirmed through analysis of culture reports of antibiotics consumed throughout the hospital specialties but we could only access limited culture isolates during study period. The latter factor (absence or non-adherence to antibiotic treatment guidelines) is evident from the rate of antibiotic use in hospital in years 2017 (78.5%) and 2018 (79.5%) which was reduced by 12.6% in the year 2019 (66.9%), most probably due to the launch of 1st version of Antimicrobial Guidelines in hospital in the same year which had an immediate effect on the percentage of antibiotics encounter but seems to be increasingly non-adherent in the post-launching years of 2020 and 2021 where again the rate of antibiotic use inclined up to 75.1% and 84.4% respectively. Although the consumption of a few antibiotics in Pakistan was higher during the years 2019 and 2020 due to COVID-19 pandemic compared to the pre-pandemic period [31]. But still overall rate of antibiotic use in this hospital is exceeding with time and this finding suggest that antibiotic prescribing in the hospital needs to be regulated, prescribers and patients belief about therapeutic efficacy of antibiotics needs to addressed, and Drug Use Evaluation (DUE) reports should be regularly performed to monitor whether the antibiotics are prescribed appropriately or not [29].

Among overall percentage of antibiotics encounter, WATCH group antibiotics were prescribed (76%) and consumed more within in-patient settings than Access (12%) and Reserve (12%) antibiotics. This proportion of AWaRe antibiotics consumption is opposite to the WHO General Program of Work 2019–2023 indicator which recommends that the proportion of Access antibiotics should be more than 60% of overall antibiotic use and Reserve antibiotics consumption should be limited for situations when all alternative antibiotics have failed. (32) This difference in AWaRe antibiotics according to WHO indicator is a huge gap and needs to be bridged by Hospital administration. This can be done by engaging the Antibiotic Stewardship team to make the healthcare team realize that, Access antibiotics represent first or second-line for empirical treatment of common infectious syndromes and have a favorable safety profile in comparison to Watch group antibiotics. Watch group antibiotics on the other hand, are among the highest priority critically important antimicrobials for human medicine recommended only for specific, limited indications but they have the higher potential to negatively impact AMR [32]. While Reserve group are “last resort” antibiotics representing a valuable, non-renewable resource that should be used as sparingly as possible [32].

In terms of magnitude measure of antibiotic consumption through RC median values, Colistin (IV) consumption in the in-patient settings increased by an average of 1798.91% from 2018 to 2021 in comparison to its negligible consumption in 2017 (taken as a baseline), which was recorded as the highest (positive) change for any antibiotic in our study. Another antibiotic such as Imipenem-Cilastatin is second in line with an average increased consumption of 1417.56%. After consulting the culture reports in the hospital settings, it was revealed that a multi-drug resistant (MDR) Gram-negative pathogen such as *Acinobacter baumani* was most common (prevalent) in hospital during the study period and Colistin was the only drug in the hospital formulary/antibiogram that was sensitive or intermediate sensitive to these bacteria at that time. This finding is consistent with highest consumption of Colistin in the hospitals of Spain and Greece where the prevalence of hospitals isolates of such pathogens was high and Colistin was used to treat MDR Gram-negative pathogens [(including *Acinetobacter* species that are resistant to Carbapenems (such as Imipenem-Cilastatin which has the second highest increased RC in our study)] [33, 34] [35]. This makes it even more clear that Colistin possibly, had also been extensively utilized to cover the strains resistant to Imipenem-Cilastatin during these years. However, this finding is suggestive of the concern and steps that seems to be missed by the Infection Control Department of the hospital to control hospital acquired pathogens (*Acinetobacter* species) from 2017 to 2021. Otherwise, RC of Colistin (which is a Reserve antibiotic and the same time nephrotoxic in nature) [36], will likely increase with an increasing occupancy rate of the hospital. Infact, in cases where Colistin resistance will be seen, another therapeutic option [(such as polymyxin B and certain tetracycline derivatives (i.e., minocycline and tigecycline)] for extensively-drug resistant *Acinetobacter* will be consumed [37, 38] and that too with an increased consumption pattern than its normal use. The same happened when we viewed our antibiotic consumption data with relevance to forementioned categories; Minocycline (a tetracycline derivative) was re-introduced in hospital in 2021 only after 2017 (and not being part of hospital formulary from 2018 to 2020) for those cases where Colistin was intermediate resistant, a peak consumption of Minocycline was seen in 2021 (an average increase of 10,851% in its consumption). The diarized key findings during consultation with clinical pharmacist working during this tenure revealed that multi drug resistance organism was increasing in hospital and an infectious disease (ID) consultant had introduced this antibiotic to cure those patients who were resistant to almost all antibiotics present in formulary. So, this shows failure to reduce hospital acquired infections can have an indirect effect in the form of enhanced consumption patterns of alternative drug of choices (i.e., antibiotics) while balancing the burden of existing resistant pathogen strains in the hospital settings.

Literature suggests two way-forwards for mitigating the antibiotic resistant bugs; one is related to the infection control team of health settings and the other is focused on hospital formulary inclusion criteria of antibiotics. The earlier approach may constitute Multi-site Gram-negative Surveillance Initiative (developed by Centre for Disease Control and Prevention) in response to growing concerns about pneumonia, bloodstream infections, wound or surgical site infections, and meningitis in healthcare and community settings [39]. This is vital because selected gram-negative bacteria are becoming resistant to all or nearly all antibiotics, leaving patients with infections from these bacteria with few or no treatment options in the future [40].

Second way-forward will be to have some check involved first, for including an antibiotic into the hospital formulary and then continuous monitoring of consumption of the included antibiotic in accordance with the rationale presented by healthcare professionals for hospital formulary inclusion for that particular antibiotic [41]. This will be helpful to develop a mechanism to identify antibiotics who are consumed in negligible proportions in initial years of inclusion in to the hospital formulary but their consumptions either jumps to an increase of several thousand folds [such as Colistin (IV) & Imipenem + Cilastatin (IV) antibiotics in our case) or they are discontinued for years and then suddenly re-introduced after years gap to accidently manage the resistant pathogen isolates (e.g., Minocycline in our study).

Gentamicin consumption has the highest negative relative change (decrease in consumption) over the study period. Its nephrotoxic effects might have been observed by clinical pharmacy team which possibly led to decrease in its use as discussed in another study [42]. After implementation of antibiotic stewardship in hospital, a decrease in consumption of Cefaclor, Ofloxacin, Streptomycin, Cefuroxime and Ciprofloxacin were observed which ultimately resulted in negative relative change. Such kind of decrease in Ceftriaxone consumption after policy implementation was seen in a study in Poland whereas DDDs of all antibiotics were 2,177.5 before and reduced to 1,335.4 after the installation of hospital antibiotic policy [43]. Moreover, a little decrease in consumption of Vancomycin (14%) was seen over the period of time which might be found out by clinical pharmacist in relation to an increase in healthcare associated infection VRE (Vancomycin-resistant Enterococcus Specie) that resulted in significantly increased consumption of Linezolid (46%) as an alternative antibiotic. The same study from Taiwan supports the following fact [44].

## Strengths

Since the proposed study site is the only and among one of the three Joint Commission International (JCI)-Accredited tertiary care hospital within Islamabad capital territory and Pakistan respectively, therefore the study results may thus add to surveillance data on national antibiotic use, which can be used to track long-term changes at the national and international levels.

Additionally, gathering data on the extent, distribution, types, and nature of antibiotic usage in hospitals may make it easier to conduct future epidemiological studies on the connection between antibiotic use and resistance.

## ﻿Limitations

Our study also has few limitations which were as follow:

- DDD/100-BDs among pediatric patient cannot be calculated properly as a complete injection dispensation will be ruled out from system where as the dose of a pediatric patient will be less than it.
- Antibiotics doses are adjusted in case of chronic kidney disease (CKD) patients according to their creatinine clearance and this cannot be rule out by DDD calculation method.
- Syrups are dispensed as unit dose and exact doses consumed from it cannot be ruled out.

## Conclusion

The hospital antibiotic consumption data is well maintained across different inpatient specialties but the mechanisms for routine monitoring of antibiotics use and optimization are still in early stages of development. Although the hospital antibiotics policy is in place but seems not to be followed with a high degree of adherence during 2017–2021 in terms of WHO AWaRe antibiotics categories; the overall percentage of encounters in which antibiotics were prescribed is exceeding by almost 47% in comparison to the WHO standard reference value, Watch antibiotics are prescribed and consumed by two-third (2/3) proportion when compared to the combined consumption of Access and Reserve group antibiotics, the mean relative change of antibiotics consumed positively are lower in number (10 vs. 14 antibiotics) but higher in proportion than antibiotics with negative relative change, a continuous variation in overall antibiotic consumption trend can be seen during a constant but slight increase in hospital occupancy rate in terms of DDD/100 BDs. The existing hospital workforce, in terms of resources and planning, is facing challenges to assess, control and implement antibiotics consumption posing an emerging threat of antibiotic resistance in coming years in the hospital.

## Future recommendations

- The impact of processes (policies and guidelines) and outcomes (improvement in patient care and antibiotic use) of hospital stewardship interventions needs to be monitored.
- The antibiotic stewardship program should prepare antibiotic resistance information (in collaboration with the hospital’s infection control, microbiology lab and healthcare epidemiology department) and regularly report this information to hospital leadership, prescribers, pharmacists and nurses.
- The hospital staff needs to be educated about optimal prescribing, adverse reactions and resistance associated with antibiotic misuse. This can be achieved through didactic presentations, poster messages, newsletters, flyers, or electronic communication.
- There should be an option for antibiotic prescribing in CPOE (Computerized physician order entry) that have the option whether the antibiotic is being prescribed for prophylactic / empiric / therapeutic purpose.
- The future studies could be focused on estimating antibiotic consumption by using days-of-therapy as an additional metric and across outpatient/ambulatory care settings.

**Table 1:** Demographics of study site

Demographic Characteristics	2017	2018	2019	2020	2021
Total Patients Admitted	34,750	36,907	44,433	34,536	42,810
Total Bed Capacity	530	530	540	550	550
Average Operational Beds	379	392	388	417	417
Bed Occupancy Rate	71%	73%	71%	75%	75%
Patient on Antibiotics	27,287	29,348	29,759	25,944	36,145
Gender Distribution on Antibiotics					
Male	15,976	17,205	16,404	15,496	21,398
Female	11,311	12,143	13,355	10,448	14,747
Percentage of encounters with antibiotics	78.5%	79.5%	66.9%	75.1%	84.4%
AWaRe classification of antibiotics					
Access antibiotics consumption	9.02%	14.93%	8.08%	12.95%	14.47%
Watch antibiotics consumption	88.21%	67.04%	72.07%	77.21%	75.38%
Reserve antibiotics consumption	2.77%	18.03%	19.86%	9.84%	10.16%

**Table 2:** Mean relative change of Access group Antibiotics

Antibiotics	Route	DDD/100-BD per Year					Mean Relative Change (%) (2017–2021)
		2017	2018	2019	2020	2021	
**Amikacin Sulphate**	IV	1.772	2.425	1.054	2.083	2.276	
Relative Change			36.830	-40.5	17.52	28.420	**10.55**
**Amoxicillin**	Oral	0.447	0.488	0.219	0.115	0.060	
Relative Change			9.23	-51.04	-74.34	-86.53	**-50.67**
**Ampicillin**	IV	0.735	1.072	0.950	0.860	0.536	
Relative Change			45.749	29.22	16.998	-27.08	**16.22**
**Benzathine Penicillin**	IM	0.001	0.0001	0.002	0.0001	0.0003	
Relative Change			-86.45	124.5	-85.41	-66.49	**-28.46**
**Benzyl Penicillin** **(penicillin G) IM/IV**	IV	0.236	0.485	0.342	0.290	0.105	
Relative Change			104.94	44.58	22.641	-55.58	**29.15**
**Co-Amoxiclav** **(Amoxicillin +** **Clavulanic acid)**	IV	5.371	5.830	3.824	7.440	3.011	
Relative Change			8.561	-28.80	38.539	-43.93	**-6.41**
**Co-Amoxiclav** **(Amoxicillin +** **Clavulanic acid)**	Oral	1.599	0.949	0.905	0.539	0.420	
Relative Change			-40.64	-43.43	-66.31	-73.74	**-56.04**
**Cefadroxil**	Oral	0.004	0.004	0.001	0.001	0.001	
Relative Change			-12.45	-83.88	-76.44	-81.95	**-63.68**
**Cefazolin**	IV	1.459	1.201	1.574	1.007	0.602	
Relative Change			-17.72	7.848	-30.95	-58.71	**-24.89**
**Cephradine**	IV	0.161	0.143	0.194	0.100	0.038	
Relative Change			-11.12	20.19	-38.18	-76.66	**-26.45**
**Cephradine**	Oral	0.060	0.156	0.020	0.022	0.007	
Relative Change			159.88	-67.16	-63.82	-87.66	**-14.69**
**Doxycycline**	Oral	0.554	0.007	0.603	1.821	0.071	
Relative Change			-98.71	8.813	228.57	-87.17	**12.87**
**Gentamicin**	IV	6.806	0.687	0.403	0.417	0.486	
Relative Change			-89.91	-94.07	-93.87	92.860	**-92.68**
**Metronidazole**	IV	5.061	5.404	6.577	5.684	2.345	
Relative Change			6.774	29.95	12.306	53.673	**-1.16**
**Trimethoprim** **/sulfamethoxazole (Co-Trimoxazole)**	Oral	0.354	0.232	0.163	0.230	0.238	
Relative Change			-34.56	-53.9	-35.04	-32.89	**-39.10**

**Table 3:** Mean relative change of Watch group Antibiotics

Antibiotics	Route	DDD/100-BD per Year					Mean Relative Change (%) (2017–2021)
		**2017**	**2018**	**2019**	**2020**	**2021**	
**Azithromycin**	IV	0.534	0.486	1.212	1.997	1.391	
Relative Change			-8.994	127.240	274.224	160.768	**138.310**
**Azithromycin**	Oral	1.445	2.768	2.097	5.362	3.067	
Relative Change			91.555	45.137	271.049	112.201	**129.986**
**Cefaclor**	Oral	0.134	0.062	0.029	0.009	0.013	
Relative Change			-53.49	-78.424	-92.905	-90.139	**-78.742**
**Cefepime**	IV	0.157	0.167	0.188	0.204	0.186	
Relative Change			6.360	19.703	29.690	18.407	**18.540**
**Cefixime**	Oral	6.013	6.378	7.560	4.497	2.559	
Relative Change			6.080	25.724	-25.207	-57.441	**-12.711**
**Cefoperazone** **+** **Sulbactam**	IV	2.022	2.352	3.476	3.616	2.138	
Relative Change			16.325	71.898	78.857	5.731	**43.203**
**Cefotaxime Sodium**	IV	0.301	0.331	0.183	0.123	0.091	
Relative Change			10.064	-39.248	-59.276	-69.707	**-39.542**
**Ceftazidime**	IV	0.207	0.296	0.401	0.390	0.523	
Relative Change			42.482	93.136	87.888	151.910	**93.854**
**Ceftraxione Sodium**	IV	24.93	23.152	18.685	17.568	16.368	
Relative Change			-7.163	-25.077	-29.555	-34.368	**-24.041**
**Cefuroxime Sodium**	IV	1.617	2.041	2.412	1.904	1.660	
Relative Change			26.206	49.112	17.743	2.668	**23.932**
**Cefuroxime Sodium**	Oral	0.049	0.031	0.021	0.008	0.017	
Relative Change			-36.10	-56.008	-83.348	-63.960	**-59.855**
**Ciprofloxacin**	IV	0.713	0.765	0.625	0.722	0.592	
Relative Change			7.266	-12.313	1.247	-16.970	**-5.193**
**Ciprofloxacin**	Oral	1.463	0.976	0.479	0.379	0.602	
Relative Change			-33.28	-67.297	-74.113	-58.832	**-58.381**
**Clarithromycin**	Oral	0.229	0.424	0.302	0.186	0.173	
Relative Change			85.394	31.973	-18.946	-24.289	**18.533**
**Ertapenem**	IV	0.469	1.072	1.211	1.418	0.900	
Relative Change			128.66	158.361	202.507	91.949	**145.370**
**Fosfomycin**	Oral	0.038	0.238	0.255	0.207	0.351	
Relative Change			531.59	576.48	448.934	829.064	**596.519**
**Imipenem** **+** **Cilastatin**	IV	0.140	0.124	3.187	3.015	2.168	
Relative Change			-11.69	2177.6	2054.58	1449.65	**1417.560**
**Levofloxacin**	IV	1.133	1.413	1.460	1.422	1.749	
Relative Change			24.681	28.840	25.467	54.318	**33.326**
**Levofloxacin**	Oral	2.596	2.207	1.557	1.631	1.467	
Relative Change			-14.99	-40.036	-37.176	-43.492	**-33.925**
**Meropenem**	IV	13.33	14.268	11.503	16.014	15.828	
Relative Change			7.010	-13.726	20.105	18.707	**8.024**
**Moxifloxacin**	IV	4.136	4.507	3.991	2.713	1.905	
Relative Change			8.964	-3.520	-34.413	-53.943	**-20.728**
**Moxifloxacin**	Oral	2.159	2.405	1.315	1.443	3.129	
Relative Change			11.378	-39.111	-33.169	44.931	**-3.993**
Minocycline*	Oral	0.016				1.772	
Relative Change						10851.8	**10851.87**
**Ofloxacin**	Oral	0.045	0.008	0.002	0.002	0.002	
Relative Change			-81.76	-94.842	-95.288	-95.790	**-91.921**
**Piperacillin** **+** **Tazobactam**	IV	10.97	8.501	6.513	8.554	6.632	
Relative Change			-22.52	-40.648	-22.047	-39.556	**-31.194**
**S** **treptomycin**	IV	0.074	0.063	0.019	0.004	0.004	
Relative Change			-14.82	-74.388	-95.274	-94.209	**-69.673**
**Teicoplanin**	IV	0.521	0.263	0.114	0.608	0.466	
Relative Change			-49.45	-78.186	16.717	-10.563	**-30.371**
**Vancomycin**	IV	14.016	14.457	10.147	11.779	10.893	
Relative Change			3.148	-27.602	-15.961	-22.280	**-15.674**

**Table 4:** Mean relative change of Reserve group antibiotics

Antibiotics	Route	DDD/100-BD per Year					Mean Relative Change (%) (2017–2021)
		2017	2018	2019	2020	2021	
Colistimethate Sodium*	IV	0.22	3.145	4.014	4.613	5.018	
Relative Change			1322.647	1715.794	1986.672	2169.7	**1798.708**
**Fosfomycin**	IV	0.475	0.376	0.496	0.596	0.344	
Relative Change			-20.861	4.437	25.508	-27.71	**-4.657**
**Linezolid**	IV	1.34	1.9100	1.930	1.88	2.140	
Relative Change			3.607	-4.454	40.391	-94.96	**46.713**
**Linezolid**	Oral	0.802	0.967	0.921	1.173	1.366	
Relative Change			20.546	14.833	46.249	70.285	**37.978**
**Tigecycline**	IV	0.550	0.458	0.514	0.513	0.322	
Relative Change			-16.796	-6.473	-6.731	-41.53	**-17.884**

## Data Availability

The datasets analyzed during the current study are available from the corresponding author upon reasonable request.
